# Partner Violence and Psychosocial Distress among Female Sex Workers in China

**DOI:** 10.1371/journal.pone.0062290

**Published:** 2013-04-23

**Authors:** Yan Hong, Chen Zhang, Xiaoming Li, Wei Liu, Yuejiao Zhou

**Affiliations:** 1 School of Rural Public Health, Texas A&M Health Science Center, College Station, Texas, United States of America; 2 Global Health Institute, Rollins School of Public Health, Emory University, Atlanta, Georgia, United States of America; 3 School of Medicine, Wayne State University, Detroit, Michigan, United States of America; 4 Guangxi Center for Disease Control and Prevention, Nanning, Guangxi, China; Old Dominion University, United States of America

## Abstract

**Background:**

Despite recognized vulnerability of female sex workers (FSW), most data on this population are focused on their HIV and STI prevalence; studies on their experience of partner violence and psychosocial distress are limited, especially FSW in China.

**Methods and Findings:**

A cross-sectional survey was administered among 1,022 FSW recruited from 9 different types of commercial sex venues in Southwest China. Partner violence scales were adapted from WHO's Women's Health and Domestic Violence scale and psychosocial distress was measured by five indicators, including alcohol intoxication, drug use, suicidal behavior, depression, and loneliness. Random effects modeling was used to control for cluster effects. Findings: About 58% of FSW ever experienced violence from their stable partners, and 45% suffered it from their clients. Partner violence was strongly associated with each of the five measures of psychosocial distress, even after controlling for potential confounders.

**Conclusion:**

This study is one of the first to examine the association between partner violence and psychosocial distress among FSW in China. The high prevalence of violence experience and distress in this population suggests urgency for intervention. The public health programs targeting FSW should go beyond the focus on HIV/STI prevention and care for the fundamental health and human rights of millions of FSW in China.

## Introduction

Violence against women is recognized as both a pervasive public health problem and a shameful human rights violation [1]–[2]. The widespread violence against women has a substantial impact on health: It leads to high rates of morbidity and mortality, including HIV and other sexually transmitted infections (STI), gynecological problems, unwanted pregnancy or abortion, depression, posttraumatic stress disorder, substance abuse, suicide and death [3]–[4]. One of most common types of violence against women is partner violence (PV), which refers to physical, sexual, or mental abuse perpetrated by the women’s husbands, boyfriends, or partners, including sexual partners such as clients of commercial sex [5]. Female sex workers (FSW) are a group most likely to be victimized by PV with documented high rates of PV in this population [6]–[7]. For example, El-Bassel et al. reported that two of every three FSW in New York experienced violence from either a stable partner or a client [8]. Shannon et al. reported that 57% of FSW in Vancouver, Canada, experienced gender-based violence including partner violence in the past 18 months [9].

Global literature documents pervasive PV against FSW especially its relationship with increased HIV risks [10]–[12]. Existing qualitative studies also depict the context and psychosocial consequences of PV [13]–[17]. Some quantitative studies have examined the relationship between PV and psychosocial consequences but they typically employed single indicator of psychosocial distress, such as substance abuse or suicidal behaviors [18]–[19]. There is a lack of studies that have applied multifaceted measures to depict different aspects of psychosocial effects of PV against FSW. Further, global literature on PV against FSW has been mostly from high-income western countries and focused on street-based, drug-using women who practice survival sex [6], [8], [9], [20]. These women are different from FSW in developing countries who primarily work in entertainment establishments or brothels and provide indirect sexual services [21]. Contrary to the popular belief that brothel-based FSW are safer than their counterparts in the streets, data revealed that street-based FSW reported more frequent physical violence; whereas brothel-based FSW were more likely to report more severe incidence of sexual violence [22]. The current study will report PV against FSW in China, where commercial sex has flourished in the past three decades.

The past 30 years have witnessed a rapid resurgence of commercial sex in China, fueled by growing income disparities, high mobility of population and changing norms of sexuality [23]. About 10 million FSW work in a complex hierarchy of commercial sex establishments [24]. Similar to existing global literature on FSW, most data on FSW in China are about their HIV and STI risks [25]. Limited data reveal that this population suffers a high frequency of PV and high rates of psychosocial distress, including suicidal behaviors. For example, Hong et al. reported that 18% of FSW had suicidal ideation and 6% had attempted suicide in the past 6 months and about two thirds of FSW had depression [26]–[27]. Despite these sporadic data, to date, no study has been conducted on the relationship between PV and psychosocial distress among FSW in China. In an attempt to fill this gap in the literature, the current study addressed the following research questions: 1) What is the prevalence of PV against FSW perpetrated by clients and stable partners respectively? And 2) how is PV associated with five measures of psychosocial distress after controlling for potential confounders?

## Methods

### Ethics Statement

The study protocol was approved by the Institutional Review Boards at the Wayne State University in the USA and Beijing Normal University in China.

### Study Site

The study was conducted in Guangxi Zhuang Autonomous Region in southwest border of China. One of the five ethnic minority autonomous regions, Guangxi is ranked the second in terms of total HIV prevalence and first in terms of new HIV infection cases [28]. Guilin City located in northeast Guangxi and Beihai City in the southern coast were selected as research sites for the study. Both cities are medium size with population over one million and both are tourists spots attracting 4–10 million visitors to each city every year. Local CDC estimated that more than 2,000 FSW worked in at least 150 commercial sex establishments in each city.

### Sampling and Participant Recruitment

We used the same sampling and recruitment strategies as we did in previous studies with FSW in China [26], [27], [29]. In the formative phase of the study, our research team worked closely with the local CDC for ethnographic mapping, in which all known commercial sex venues were updated on the maps. Participants in the current study were recruited from 60 establishments (31 in Guilin and 29 in Beihai) that represented all known commercial sex venues in Beihai and Guilin, including saunas, night clubs, bars, karaoke (KTV), bars, massage parlors, hair salons, restaurants, mini hotels, and the streets. We first contacted the owners or managers of each representing establishment for permission to conduct research on their premises; if they declined (less than 30%), we identified other venues of the same type and similar size until the targeted sample size (i.e., approximately 500 FSWs in each city) was achieved. Once we obtained permission from the manager, we invited FSW in the site to participate; about 20% of FSW in participating venues declined to participate.

### Data Collection Procedure

We conducted the survey in separate rooms or private spaces in the establishments; only the interviewer and the participant were allowed to stay in the room. After the informed consent, the participant completed a structured questionnaire in a paper-pencil format; the interviewer only provided assistance when necessary. It took about 45 minutes to complete the questionnaire and each participant received a small gift equivalent to 30 yuan ($4.5). Among 1,022 women who completed the survey, 937 provided information on violence from clients and were included in the analysis; 279 women did not report having stable partners and therefore did not provide data on PV from stable partners.

### Measures

***Demographic information*** collected in the survey include age, ethnicity (Han or ethnic minority), residency (rural or urban household registration), education (completed middle school or not), marital status (ever married or not), length of migration (months of living in the city), length of working in the city (months of working in the city), living with partners or not, working venue, and monthly income. Because of high correlation between length of migration and length of working, we only included in the latter in the data analysis. Because of the hierarchy of commercial sex establishments, the venues were categorized into four types by the mean income of FSWs at each venue: Level 1 included those venues with mean income higher than 3,000 yuan each month (in this study, only FSWs working sauna had a mean income higher than 3,000 yuan); Level 2 consisted of those venues with mean income between 2,000 to 3,000 yuan (night club, KTV, bar, dancing halls); Level 3 included those venues with mean income between 1,000 to 2,000 yuan (message parlor, hair salon); and Level 4 consisted of those venues with mean income less than 1,000 yuan (restaurant, mini hotel, and streets).

***Partner violence*** scales were adapted from the WHO’s Multi-Country Study on Women’s Health and Domestic Violence [1]. PV measured in the current study covered three dimensions: physical violence (e.g., slapped you or threw something at you that could hurt you; pushed you or shoved you or pulled your hair; kicked you; dragged you or beat you up), sexual violence (e.g., forced you into intercourse; inserted something into your genitals), and psychological abuse (e.g., belittled or humiliated you in front of others; threatened to hurt you or someone you care about). PV from stable partners included three additional items, for example, ignoring you for a long time, threat of separating you from your children or terminating your pregnancy, and restriction of your freedom. All the items were assessed using a 4-point scale: 0 = never, 1 = occasionally, 2 = sometimes, and 3 = frequently. The PV from clients included 17 items with a composite score ranging from 0 to 51; the PV from stable partners included 20 items with a composite score ranging 0 to 60. Because of big range and skewed distributions of both variables, we used binary score (dichotomized never or ever experience PV) in data analysis.

***Psychosocial distress*** was measured by five indicators: alcohol intoxication, drug use, suicidal behavior, depression, and loneliness; all indicators were validated in Chinese language including our own studies of FSW in China [26], [27], [29]. A*lcohol intoxication* was measured by frequency of alcohol intoxication (e.g., every day, once every 2–3 days, once a week, once every 2–3 weeks, never). Respondents were categorized into never or ever had abuse intoxication. *Drug use* was measured by a single item: “Have you ever used illicit drugs? (Yes/No)”; respondents were categorized into never or ever groups. *Suicidal behavior* was measured by two items of suicidal ideation (“had seriously considered killing yourself”) and suicidal attempt (“had tried to kill yourself”). Respondents who answered “yes” to either of these two questions were categorized into ever had suicidal behavior and the rest into never. *Depression* was measured by the Center for Epidemiologic Studies Depression scale (CES-D) [30], which was validated in Chinese culture.^31^ The Cronbach’s alpha for the current study sample was.89; and the mean score was 17.35 (ranged 0 to 53); a cut-off point of 16 was used for dichotomization [31]. *Loneliness* was measured by the UCLA Loneliness scale [32]. The Cronbach’s alpha of the scale in the current study was.74; the mean score was 43.30 (ranging from 20 to 75); a cut-off point of 44 was used for dichotomization [32].

### Data Analysis

First, we used Chi-square and *t*-test to compare demographic characteristics between those who had never and ever experienced PV from stable partners and clients. Second, multivariate logistic regression models were built to examine the independent relationship between PV and key demographic variables. To control for potential intra-class correlation (ICC) by venue due to cluster-sampling, we used random effect modeling. Adjusted odds ratio (aOR) and 95% confidence intervals (95% CI) were used to depict an independent relationship between dependent and independent variables. Third, the association between PV and each of the five indicators of psychosocial distress was analyzed using Chi-square or *t*-test. Finally, multivariable regression models were built to examine further the relationship between PV and psychosocial distress (each of the five indicators and the total psychosocial distress score) while controlling for potential confounders. Variables of significance in Step 2 were included in the multivariable models. Similar to Step 2, random-effect modeling was employed to control for ICC; adjusted odds ratio (aOR) for logistic regression models and 95% CI were used to depict the independent relationship between dependent and independent variables. Data management and analysis were performed using Stata 10.0.

## Results

### Demographic Characteristics and Partner Violence

As shown in Table 1, the mean age of the participants was 24.9. Most of them were Han, more than half of them were from rural areas, and 72% were never married. They had worked in the cities on an average of 44 months and earned 2,660 yuan a month; the income varied considerably among individuals and across different venues. About 45% FSW (422 out of 937) ever experienced PV from their clients; among FSWs who had stable partners, 58% (430 out of 743) ever experienced PV from their stable partners. Roughly 21% FSW experienced PV from stable partners only and 20% experienced PV from clients only; nearly 20% had PV from both stable partner and clients (data not shown in table).

**Table 1:** Demographic characteristics of FSWs and relationship with Partner Violence.

	Total (n = 1022)	Violence from Stable Partners (n = 743)^1^		Violence from Clients (n = 937)^2^	
		Never (n = 313)	Ever (n = 430)	Never (n = 515)	Ever (n = 422)
**Age,** Mean (SD)	24.89 (6.67)	25.81(6.62)	24.88(6.95)	24.78(6.74)	24.86(6.68)
**Ethnicity**					
Han	84.4%	89.50%	84.00%*	87.20%	81.50%*
Non-Han	15.6%	10.50%	16.00%	12.80%	18.50%
**Residency**					
Urban	44.4%	46.90%	42.30%	43.90%	44.80%
Rural	55.6%	53.10%	57.70%	56.10%	55.20%
**Education**					
<middle schl	63.4%	58.80%	69.10%***	63.30%	65.20%
≥middle schl	36.6%	41.20%	30.90%	36.70%	34.80%
**Marital Status**					
Never	71.5%	64.90%	70.00%	71.80%	72.00%
Ever	28.5%	35.10%	30.00%	28.20%	28.00%
**Length of Working** mean (sd)	43.98 (35.81)	47.19 (39.05)	44.65 (34.94)	41.47 (33.48)	45.27 (37.46)
**Living arrangement**					
Living with Partner	27.4%	25.93%	27.59%	25.93%	27.59%
Not living w. partner	72.6%	74.07%	72.41%	74.07%	72.41%
**Venue Level** 3					
Level 1	27.00%	23.29%	24.81%	25.34%	33.74%*
Level2	57.00%	58.22%	58.48%	57.96%	50.00%
Level3	7.2%	8.56%	5.82%	7.27%	7.64%
Level4	8.7%	9.93%	10.89%	9.43%	8.62%
**Income** RMB^4^, mean (sd)	2667.52 (2364.20)	2507.92 (2379.10)	2591.60 (2178.01)	2767.41 (2480.32)	2682.00 (2361.79)

Note: ^1^: FSWs who reported having stable partners, and who completed more than half of the 20-item IPV-stable partner scale.

2 FSWs who completed more than half of the 17-item IPV-client scale.

3 Venues were grouped based on the median income of FSWs in each venue. Level 1 refers to sauna (mean income higher 3,000 RMB/month); Level 2 refers to Karaoke, bars, dancing halls, and night clubs (mean income between 2,000 and 3,000 RMB/month); Level 3 refers to hair washing room, massage parlor, and restaurants (mean income between 1,000 and 2,000 RMB/month); Level 4 refers to mini hotels and streets (mean income less than 1,000 RMB/month). Also see Methods section for details.

4 At the time of study, the currency exchange rate for RMB and USD was: 1 USD = 6.3 RMB.

* p<.05,

*** p<.005.

As shown in Table 2, among FSW ever experienced PV from stable partners, PV was significantly associated with ethnic minority and rural residency, having less than middle school education, and living with stable partners. Among FSW who ever experienced PV from clients PV was significantly associated with ethnic minority, and working in lower level of commercial sex venues.

**Table 2:** Multivariate logistic regression of partner violence on key demographic characteristics^1^.

	Violence from Stable Partners (n = 743)	Violence from Clients (n = 937)
	aOR (95% CI)	aOR (95% CI)
**Age,**	0.96 (0.92, 1.00)	1.0 (0.96, 1.04)
**Non-Han ethnicity**	1.91 (1.10, 2.98)*	1.54 (1.04, 2.26)*
**Residency**	0.97 (0.70, 1.36)	0.89 (0.66, 1.19)
**Had ≥middle school educ.**	0.55 (0.39, 0.77)***	0.87 (0.64, 1.18)
**Have been married**	0.88 (0.56, 1.39)	0.98 (0.64, 1.52)
**Living w stable partners**	1.55 (1.11, 2.16)*	1.09 (0.80, 1.50)
**Venue Level** 2		
Level1	**Reference**	**Reference**
Level2	0.91 (0.61, 1.34)	0.64 (0.43, 0.96)*
Level3	0.71 (0.34, 1.46)	0.80 (0.40, 1.59)
Level4	1.82 (0.81, 4.11)	0.84 (0.34, 2.05)
**Income**	1.0 (0.93, 1.07)	1.0 (0.94, 1.06)

Note: ^1^Random effect model, justifying intra-class correlation within each venue due to cluster sampling.

2 Venues were grouped based on the median income of FSWs in each venue.

* p<.05,

*** p<.005.

### Association of Partner Violence and Psychosocial Distress

PV was significantly and positively associated with all of the five indicators of psychosocial distress. For instance, 56% of FSW who had PV from stable partners had depression compared to 40% in their counterparts; 70% of participants experienced PV from stable partner reported alcohol intoxication compared to 56% in their counterparts; and 12.1% of respondents who ever had PV from stable partners had suicidal behavior, compared with 4.8% of their counterparts.

The mean score of CED-S among FSWs who had ever experienced PV from stable partner was as high as 17.9(SD = 9.2), compared to 14.8(SD = 9.0) in their counterparts. For those who had experienced PV from clients, psychosocial distresses included suicidal behaviors, drug abuse, depression, and loneliness. Women who reported violence from clients also had more elevated CES-D scores (mean = 18.6) than their counterparts (mean = 16.3). About 13% of FSW who had experienced PV from clients had suicidal behaviors, compared to 7.2% of their counterparts (Table 3).

**Table 3:** Association between Partner Violence and psychosocial distress.

		Violence from Stable Partners (n = 743)		Violence from Clients (n = 937)	
	Total (n = 1022)	Never (n = 313)	Ever (n = 430)	Never (n = 515)	Ever (n = 422)
**Depression** (CES-D) (mean, SD)	17.12 (9.65)	14.82 (9.00)	17.94 **** (9.16)	16.32 (9.54)	18.64*** (9.74)
** Depression,^1%^**	49.05%	39.73%	55.95%****	47.35%	59.61%****
**Loneliness** (mean, SD)	43.62 (7.87)	41.23 (7.79)	44.44**** (7.24)	42.58 (7.81)	44.54**** (7.64)
** Loneliness,^2%^**	47.31%	36.99%	54.94%****	46.37%	56.16%***
**Alcohol intoxication,** %	67.26%	56.5%	70.00%****	59.20%	65.40%*
**Drug abuse,** %	18.30%	15.30%	20.70%*	15.50%	23.20%***
**Suicidal behavior**, %	9.50%	4.80%	12.10%****	7.20%	13.0%***

1 Cut-off point for depression scale: 16.

2 Cut-off point for loneliness scale: 44.

* p<.05,

*** p<.005,

**** p<.0001.

As shown in the Tables 4 and 5, even after controlling for potential confounders, PV from stable partner were still significantly associated with depression (aOR = 2.00), loneliness (aOR = 2.08), alcohol intoxication (aOR = 2.27), and suicidal behavior (aOR = 2.91) (Table 4). Likewise, PV from clients was significantly associated with depression (aOR = 1.76), loneliness (aOR = 1.61), alcohol intoxication (aOR = 1.87), drug abuse (aOR = 1.99), and suicidal behavior (aOR = 1.93) (Table 5). Other risk factors for psychosocial distress included younger age and working in lower-end commercial sex venues.

**Table 4:** Multivariate regression^1^ of psychosocial distress indicators on partner violence from stable partners (n = 743)^1^.

	Depression	Loneliness	alcohol intoxication	Drug abuse	Suicide
	aOR (95% CI)	aOR (95% CI)	aOR (95% CI)	aOR (95% CI)	aOR (95% CI)
**Experience violence from stable partner**	2.00**** (1.45, 2.76)	2.08**** (1.51, 2.87)	2.27**** (1.52, 3,39)	1.26 (0.80, 1.99)	2.91*** (1.54, 5.49)
**Age**	1.0 (0.96, 1.03)	0.96* (0.92, 0.99)	0.97 (0.93, 1.02)	0.81**** (0.76, 0.87)	0.95 (0.89, 1.01)
**Non-Han ethnicity**	0.85 (0.53, 1.36)	0.84 (0.53, 1.34)	0.91 (0.50, 1.64)	1.09 (0.60, 1.98)	1.21 (0.60, 2.46)
**Had ≥middle school educ.**	0.93 (0.67, 1.31)	1.11 (0.80, 1.56)	1.33 (0.87, 2.02)	1.09 (0.70, 1.72)	1.57 (0.89, 2.75)
**Living w. stable partners**	0.74 (0.53, 1.02)	0.90 (0.65, 1.25)	1.00 (0.67, 1.50)	0.98 (0.62, 1.56)	0.60 (0.33, 1.09)
**Venue level** 2					
Level1	**Reference**	**Reference**	**Reference**	**Reference**	**Reference**
Level2	1.70** (1.24, 2.53)	1.60* (1.09, 2.36)	6.15**** (3.4, 11.15)	1.87 (1.00, 3.48)	3.02* (1.30, 7.04)
Level3	0.85 (0.41, 1.78)	1.38 (0.67, 2.81)	0.72 (0.27, 1.92)	2.65 (0.84, 8.36)	4.51* (1.24, 16.4)
Level 4	2.07 (0.88, 4.87)	3.30*** (1.47, 7.37)	0.43 (0.12, 1.48)	1.47e-07 (0, ∼)	2.12 (0.3, 14.58)
**Income**	0.98 (0.21, 2.50)	0.99 (0.92, 1.06)	0.92 (0.84, 1.01)	1.04 (0.94, 1.15)	1.06 (0.94, 1.20)

Note: ^1^Random effect model, justifying intra-class correlation within each venue due to cluster sampling.

2 Venues were grouped based on the median income of FSWs in each venue.

* p<.05,

** p<.01,

*** p<.005,

**** p<.0001.

**Table 5:** Multivariate regression^1^ of mental health indicators on partner violence from clients (N = 937)^1^.

	Depression	Loneliness	alcohol intoxication	Drug abuse	Suicidal behavior
	aOR (95% CI)	aOR (95% CI)	aOR (95% CI)	aOR (95% CI)	aOR (95% CI)
**Experience violence from clients**	1.76**** (1.34, 2.30)	1.61*** (1.22, 2.11)	1.87*** (1.31, 2.65)	1.99**** (1.36, 2.91)	1.93*** (1.23, 3.03)
**Age**	0.99 (0.96, 1.02)	0.96 (0.93, 0.99)	0.97 (0.93, 1.00)	0.84**** (0.80, 0.89)	0.96 (0.92, 1.01)
**Non-Han ethnicity**	1.11 (0.76, 1.60)	0.82 (0.56, 1.18)	1.34 (0.84, 2.16)	0.97 (0.59, 1.570	1.34 (0.77, 2.33)
**Had >middle school educ.**	0.92 (0.69, 1.22)	1.00 (0.76, 1.34)	1.13 (0.79, 1.63)	1.08 (0.74, 1.59)	1.17 (0.73, 1.85)
**Venue level** 2					
Level 1	**Reference**	**Reference**	**Reference**	**Reference**	**Reference**
Level2	1.95**** (1.42, 2.67)	1.68*** (1.18, 2.40)	7.94**** (4.43, 14.2)	2.34*** (1.29, 4.26)	1.68 (0.97, 2.92)
Level3	0.92 (0.52, 1.63)	1.47 (0.80, 2.71)	0.81 (0.33, 1.99)	3.00* (1.09, 8.22)	2.18 (0.88, 5.45)
Level 4	2.48 (1.23, 5.02)	2.61* (1.19, 5.71)	0.53 (1.53, 1.80)	0.0001 (0, ∼)	1.08 (0.24, 4.81)
**Income**	1.01 (0.95, 1.07)	0.99 (0.93, 1.04)	0.97 (0.90, 1.04)	1.07 (0.99, 1.16)	1.07 (0.99, 1.17)

Note:^ 1^Random effect model, justifying intra-class correlation within each venue due to cluster sampling.

2 Venues were grouped based on the median income of FSWs in each venue, see Methods section for details.

* p<.05,

*** p<.005,

**** p<.0001.

## Discussion

Several limitations should be noted before we interpret the study findings. First, our study was conducted in Guangxi, a multiethnic region in Southwest China. Current findings may not be generalizable to other areas of China. Second, we might have over sampled FSWs working in higher-income commercial sex venues. Our sampling scheme was based on a map of commercial sex venues maintained by the local centers for disease control and prevention. The map was updated during our formative phase of the project. It might be that women in lower levels of the commercial sex hierarchy were less visible (most did not have regular venues) and therefore were not identified in our ethnographic mapping. However, the demographic characteristics of the current sample were consistent with other studies of FSWs in urban areas of China [25]. Third, we did not analyze different types of PV (i.e., sexual, physical and mental violence) perpetrated by clients and stable partners, the effects of different types of PV on psychosocial distress merit further research. Fourth, our study was based on a cross-sectional design, which precluded us from drawing any causal conclusions. Though some existing literature suggests that exposure to violence caused higher level of psychosocial distress [3], [9]; the relationship could also be reverse or reciprocal [33]. Fifth, the psychosocial distress indicators included in the current study were limited; some were only single-item measures. Furthermore, our study didn’t include stressful life events (e.g., childhood sexual abuse, sex work-related stigma, and incarceration) that may contribute to FSWs’ psychosocial distress [13], [34], [35]. Finally, like all other community-based studies, our data were subject to volunteer bias and social desirability bias.

Despite these limitations, the current study represents one of the first efforts to investigate PV perpetuated by stable partners and clients against FSWs and its effects on their psychosocial distress. Our data revealed a high prevalence of PV experienced by FSW in China; PV was independently and significantly associated with key indicators of psychosocial distress. The prevalence of PV experienced by FSW in China is lower than among street-based FSW in Western countries [8], [9], but much higher than the PV experienced by Chinese women in general (10–38%) [36], [37]. Although previous qualitative studies documented the negative effects of PV experienced by FSW, our study provided quantitative data on the association between PV and five indicators of psychosocial distress.

Our findings revealed that younger age, minority, lower education, and living with partners were associated with higher rates of PV, which is similar to previous studies on FSW in other countries. For example, higher rates of PV were reported by aboriginal or native FSW in Canada, minority FSW in the USA, younger women with less education in other Asian countries [8], [10], [17]. Such evidence suggests that certain groups of FSW are more vulnerable to PV and deserve more attention and special assistance.

Previous researchers have also documented that stable partners were a constant source of stress and major perpetrators of violence against FSW [14], [34]. For instance, a study with FSW in China reported that FSW with stable partners were twice as likely to have suicidal behaviors compared to those without stable partners [26]. In the current study, we observed a higher frequency of PV perpetrated by stable partners; such PV was also linked to more detrimental effects, such as higher rates of alcohol intoxication and suicidal behaviors. In spite of their significant role in FSW’s life, FSW’s stable partners have rarely been included in health promotion programs targeting FSW.

PV has long been recognized in the literature but inadequately addressed in public health programs [38], [39]. Violence against FSW is related to cultural and religious taboos associated with female sexuality and the sale of sex [40]. Throughout the world, these cultural taboos have become institutionalized by defining sex work as criminal behavior, with enforcement of sanctions directed more often toward FSW than their clients [41]. The stable partners, clients, and the society as a whole consider FSW and their work not only illegal, but immoral and perhaps deserving of punishment [42]. In China, the oppressive patriarchal social structure and the cultural sanctions on “impurity” are aggravated by the increasing gender inequality as a result of uneven economic growth and rapid social transition [23], [43]. In this sense, the PV observed in the current study is a small reflection of the “institutional violence” or “structural violence” against FSW [44].

Global recognition of PV against women and the discussion of policies and programs to address this problem rely on an assumption of legal status of these women or focus on the general female population [1], [38], [39]. FSW, because of their illegal or immoral status, have typically been omitted from the discussion despite the sheer number of this population and the high prevalence and severity of PV they experience. Most studies and discussion of FSW, however, have typically taken place within the discourse of “HIV prevention”; even the advocacy of decriminalization or legalization of commercial sex was driven by the goal of HIV prevention [40]. We acknowledge huge progress on women’s health and human rights in the global combat of HIV, but call for more attention to the wellbeing of FSW and a break in the vicious circle of violence that is perpetrated by inequality of the economic and oppressive social and political reality of women’s lives.

Interventions to break this vicious circle can be carried out at the micro or community level and at the macro or policy level [17]. At the micro level, male partners and clients must be included in violence reduction programs; they and other members of the society should be aware of the accountability and responsibility for the outcomes of violence. We also need to provide counseling services to FSW [14]; these services can be integrated into the existing voluntary counseling and testing (VCT) clinics, a service network structure available in most of urban centers and townships in China [45]. At the macro level, we advocate for decriminalization of sex work, so that FSW could seek help when they are subject to violence. We also call for more education and employment opportunities for women, especially for young women from rural areas. Deprivation of alternative employment and lack of opportunities for advancement in life have been the main reasons for seeking work in the commercial sex [46].

Our data underscore an urgency to reduce PV perpetuated by clients and stable partners against FSWs. In China, the 10 million FSW represent a large number of young women; their psychosocial wellbeing and basic health and human rights deserve more attention and public health efforts at community, national, and international levels.

## References

[1] Garcia-Moreno C, Jansen HA, Ellsberg M, Heise L, Watts CH (2006) Prevalence of intimate partner violence: findings from the WHO multi-country study on women's health and domestic violence. Lancet 368: 1260–1269. 17027732 10.1016/S0140-6736(06)69523-8

[2] Watts C, Zimmerman C (2002) Violence against women: global scope and magnitude. The Lancet 359: 1232–1237. 10.1016/S0140-6736(02)08221-1 11955557

[3] Campbell JC (2002) Health consequences of intimate partner violence. Lancet 359: 1331–1336. 11965295 10.1016/S0140-6736(02)08336-8

[4] Tjaden P, Thoennes N (2000) Extent, nature, and consequences of intimate partner violence: Findings from the National Violence Against Women Survey: National Institute of Justice Washington, DC.

[5] Krantz G, Garcia-Moreno C (2005) Violence against women. J Epidemiol Community Health 59: 818–821. 16166351 10.1136/jech.2004.022756 PMC1732916

[6] Church S, Henderson M, Barnard M, Hart G (2001) Violence by clients towards female prostitutes in different work settings: questionnaire survey. BMJ 322: 524. 11230067 10.1136/bmj.322.7285.524 PMC26557

[7] Goodyear MDE, Cusick L (2007) Protection of sex workers. BMJ 334: 52. 17218668 10.1136/bmj.39087.642801.BE PMC1767242

[8] El-Bassel N, Witte SS, Wada T, Gilbert L, Wallace J (2001) Correlates of partner violence among female street-based sex workers: substance abuse, history of childhood abuse, and HIV risks. AIDS Patient Care STDS 15: 41–51. 11177587 10.1089/108729101460092

[9] Shannon K, Kerr T, Strathdee SA, Shoveller J, Montaner JS, et al (2009) Prevalence and structural correlates of gender based violence among a prospective cohort of female sex workers. BMJ 339: b2939. 19671935 10.1136/bmj.b2939 PMC2725271

[10] Choi SY, Chen KL, Jiang ZQ (2008) Client-perpetuated violence and condom failure among female sex workers in southwestern China. Sex Transm Dis 35: 141–146. 17921913 10.1097/OLQ.0b013e31815407c3

[11] Dunkle KL, Jewkes RK, Brown HC, Gray GE, McIntryre JA, et al (2004) Gender-based violence, relationship power, and risk of HIV infection in women attending antenatal clinics in South Africa. Lancet 363: 1415–1421. 15121402 10.1016/S0140-6736(04)16098-4

[12] Maman S, Campbell J, Sweat MD, Gielen AC (2000) The intersections of HIV and violence: directions for future research and interventions. Soc Sci Med 50: 459–478. 10641800 10.1016/s0277-9536(99)00270-1

[13] Jeal N, Salisbury C, Turner K (2008) The multiplicity and interdependency of factors influencing the health of street-based sex workers: a qualitative study. Sex Transm Infect 84: 381–385. 18596067 10.1136/sti.2008.030841

[14] Karandikar S, Prospero M (2010) From client to pimp: male violence against female sex workers. J Interpers Violence. 2010 252: 257–273. 10.1177/0886260509334393 19553559

[15] Bilardi JE, Miller A, Hocking JS, Keogh L, Cummings R, et al (2011) The job satisfaction of female sex workers working in licensed brothels in Victoria, Australia. J Sex Med 81: 116–122. 10.1111/j.1743-6109.2010.01967.x 20722786

[16] Rhodes T, Simic M, Baros S, Platt L, Zikic B (2008) Police violence and sexual risk among female and transvestite sex workers in Serbia: qualitative study. BMJ 337: a811. 18667468 10.1136/bmj.a811 PMC2492575

[17] Shannon K, Kerr T, Allinott S, Chettiar J, Shoveller J, et al (2008) Social and structural violence and power relations in mitigating HIV risk of drug-using women in survival sex work. Soc Sci Med 66: 911–921. 18155336 10.1016/j.socscimed.2007.11.008

[18] Rossler W, Koch U, Lauber C, Hass AK, Altwegg M, et al (2010) The mental health of female sex workers. Acta Psychiatr Scand 122: 143–152. 20105147 10.1111/j.1600-0447.2009.01533.x

[19] Shahmanesh M, Wayal S, Cowan F, Mabey D, Copas A, et al (2009) Suicidal behavior among female sex workers in Goa, India: the silent epidemic. Am J Public Health 99: 1239–1246. 19443819 10.2105/AJPH.2008.149930 PMC2696657

[20] Surratt HL, Inciardi JA (2010) An effective HIV risk-reduction protocol for drug-using female sex workers. J Prev Interv Community 38: 118–131. 20391059 10.1080/10852351003640732 PMC2879022

[21] Harcourt C, Donovan B (2005) The many faces of sex work. Sex Transm Infect 81: 201. 15923285 10.1136/sti.2004.012468 PMC1744977

[22] Raphael J, Shapiro DL (2004) Violence in indoor and outdoor prostitution venues. Violence Against Women 10: 126.

[23] Gil VE, Wang MS, Anderson AF, Lin GM, Wu ZO (1996) Prostitutes, prostitution and STD/HIV transmission in mainland China. Soc Sci Med 42: 141–152. 8745115 10.1016/0277-9536(95)00064-x

[24] Huang Y, Henderson GE, Pan S, Cohen MS (2004) HIV/AIDS risk among brothel-based female sex workers in China: Assessing the terms, content, and knowledge of sex work. Sex Transm Dis 31: 695. 15502679 10.1097/01.olq.0000143107.06988.ea

[25] Hong Y, Li X (2008) Behavioral studies of female sex workers in China: A literature review and recommendation for future research. AIDS Behav 12: 623–636. 17694431 10.1007/s10461-007-9287-7

[26] Hong Y, Li X, Fang X, Zhao R (2007) Correlates of suicidal ideation and attempt among female sex workers in China. Health Care Women Int 285: 490–505. 10.1080/07399330701226529 PMC1934508 17469002

[27] Hong Y, Li X, Fang X, Zhao R (2007) Depressive symptoms and condom use with clients among female sex workers in China. Sex health 4: 99–104. 17524287 10.1071/sh06063 PMC1941656

[28] Guangxi CDC (2009) Updates on HIV/AIDS Epidemic in Guangxi. Workshop of NIAAA Venue-Based HIV and Alcohol Risk Reduction among Female Sex Workers in China. Guilin, Guangxi, China.

[29] Hong Y, Fang X, Li X, Liu Y, Li M, et al (2010) Self-perceived stigma, depressive symptoms, and suicidal behaviors among female sex workers in China. J Transcult Nurs 21: 29–34. 19820172 10.1177/1043659609349063 PMC8185878

[30] Radloff LS (19770 The CES-D scale A self-report depression scale for research in the general population. Appl Psych Meas 1: 385.

[31] Lin N (1989) Measuring depressive symptomatology in China. J Nerv Ment Dis 177: 121–131. 2918295 10.1097/00005053-198903000-00001

[32] Russell D, Peplau LA, Ferguson ML (1978) Developing a measure of loneliness. J Pers Assess 42: 290–294. 660402 10.1207/s15327752jpa4203_11

[33] Kilpatrick DG, Acierno R, Resnick HS, Saunders BE, Best CL (1997) A 2-year longitudinal analysis of the relationships between violent assault and substance use in women. J Consult Clin Psych 65: 834. 10.1037//0022-006x.65.5.834 9337502

[34] Reed E, Gupta J, Biradavolu M, Devireddy V, Blankenship KM (2010) The context of economic insecurity and its relation to violence and risk factors for HIV among female sex workers in Andhra Pradesh, India. Public Health Rep 125: S81–89. 10.1177/00333549101250S412 PMC2882978 20629253

[35] Sanders T (2004) A continuum of risk? The management of health, physical and emotional risks by female sex workers. Sociol Health Ill 26: 557–574. 10.1111/j.0141-9889.2004.00405.x 15283777

[36] Parish WL, Wang T, Laumann EO, Pan S, Luo Y (2004) Intimate partner violence in China: national prevalence, risk factors and associated health problems. Int Fam Plan Perspect 30: 174–181. 15590383 10.1363/3017404

[37] Xu X, Zhu F, O'Campo P, Koenig MA, Mock V, et al (2005) Prevalence of and risk factors for intimate partner violence in China. Am J Public Health 95: 78–85. 15623864 10.2105/AJPH.2003.023978 PMC1449856

[38] Garcia-Moreno C, Heise L, Jansen HA, Ellsberg M, Watts C (2005) Public health. Violence against women. Science 310: 1282–1283. 16311321 10.1126/science.1121400

[39] Garcia-Moreno C, Watts C (2011) Violence against women: an urgent public health priority. Bull World Health Organ 89: 2. 21346880 10.2471/BLT.10.085217 PMC3040024

[40] Shannon K, Csete J (2010) Violence, condom negotiation, and HIV/STI risk among sex workers. JAMA 304: 573–574. 20682941 10.1001/jama.2010.1090

[41] Rekart ML (2006) Sex-work harm reduction, Lancet. 366: 2123–2134. 10.1016/S0140-6736(05)67732-X 16360791

[42] Ratinthorn A, Meleis A, Sindhu S (2009) Trapped in circle of threats: violence against sex workers in Thailand. Health Care Women Int 30: 249–269. 19191121 10.1080/07399330902733281

[43] Hershatter G (1996) Sexing modern China. Remapping China-Fissures in Historical Terrain. Stanford University Press; CA: 77–96.

[44] Brooks-Gordon B (2008) State violence towards sex workers. BMJ 37: a908. 10.1136/bmj.a908 18667469

[45] Wu Z, Wang Y, Mao Y, Sullivan SG, Juniper N, et al (2011) The integration of multiple HIV/AIDS projects into a coordinated national programme in China. B World Health Organ 89: 227–233. 10.2471/BLT.10.082552 PMC3044250 21379419

[46] Zheng T (2009) Red lights: The lives of sex workers in postsocialist China: University of Minnesota Press.

